# Genome-wide association study of pediatric obsessive-compulsive traits: shared genetic risk between traits and disorder

**DOI:** 10.1038/s41398-020-01121-9

**Published:** 2021-02-02

**Authors:** Christie L. Burton, Mathieu Lemire, Bowei Xiao, Elizabeth C. Corfield, Lauren Erdman, Janita Bralten, Geert Poelmans, Dongmei Yu, S.-M. Shaheen, Tara Goodale, Vanessa M. Sinopoli, OCD Working Group of the Psychiatric Genomics Consortium, Kathleen D. Askland, Cristina Barlassina, O. Joseph Bienvenu, Donald Black, Michael Bloch, Helena Brentani, Beatriz Camarena, Carolina Cappi, Danielle Cath, M. Cristina Cavallini, Valentina Ciullo, David Conti, Edwin H. Cook, Vladimir Coric, Bernadette A. Cullen, Danielle Cusi, Lea K. Davis, Richard Delorme, Damiaan Denys, Eske Derks, Valsamma Eapen, Christopher Edlund, Peter Falkai, Abby J. Fyer, Daniel A. Geller, Fernando S. Goes, Hans J. Grabe, Marco A. Grados, Benjamin D. Greenberg, Edna Grünblatt, Wei Guo, Ana G. Hounie, Michael Jenike, Clare L. Keenan, James L. Kennedy, Ekaterina A. Khramtsova, James A. Knowles, Janice Krasnow, Cristoph Lange, Nuria Lanzagorta, Marion Leboyer, Kung-Yee Liang, Christine Lochner, Fabio Macciardi, Brion Maher, Carol A. Mathews, Manuel Mattheisen, James T. McCracken, Nathaniel McGregor, Nicole C. R. McLaughlin, Euripedes c. Miguel, Benjamin Neale, Gerald Nestadt, Paul S. Nestadt, Humberto Nicolini, Erika L. Nurmi, Lisa Osiecki, John Piacentini, Christopher Pittenger, Danielle Posthuma, Ann E. Pulver, Steven A. Rasmussen, Scott Rauch, Margaret A. Richter, Mark A. Riddle, Stephan Ripke, Stephan Ruhrmann, Aline S. Sampaio, Jack F. Samuels, Jeremiah M. Scharf, Yin Yao Shugart, Jan H. Smit, Dan J. Stein, S. Evelyn Stewart, Maurizio Turiel, Homero Vallada, Jeremy Veenstra-VanderWeele, Nienke Vulink, Michael Wagner, Susanne Walitza, Ying Wang, Jens Wendland, Gwyneth Zai, Noam Soreni, Gregory L. Hanna, Kate D. Fitzgerald, David Rosenberg, Gerald Nestadt, Andrew D. Paterson, Lisa J. Strug, Russell J. Schachar, Jennifer Crosbie, Paul D. Arnold

**Affiliations:** 1 Neurosciences and Mental Health, Toronto, Canada; 2 Genetics and Genome Biology Hospital for Sick Children, Toronto, Canada; 3 Department of Human Genetics, Radboud University Medical Center, Nijmegen, the Netherlands; 4 Donders Institute for Brain, Cognition and Behaviour, Nijmegen, The Netherlands; 5 The Psychiatric and Neurodevelopmental Genetics Unit, Center for Genomic Medicine, Department of Psychiatry, Massachusetts General Hospital, Boston, MA USA; 6 The Stanley Center for Psychiatric Research, Broad Institute of MIT and Harvard, Cambridge, MA USA; 7 The Mathison Centre for Mental Health Research and Education, Hotchkiss Brain Institute, Calgary, Canada; 8 Departments of Psychiatry and Medical Genetics, Cumming School of Medicine, University of Calgary, Calgary, Canada; 9 Institute of Medical Science, University of Toronto, Toronto, Canada; 10 Department of Psychiatry and Behavioural Neurosciences, McMaster University, Ontario, Canada; 11 Department of Psychiatry, University of Michigan, Ann Arbor, MI USA; 12 Department of Psychiatry and Behavioural Neurosciences, Wayne State University, Detroit, MI USA; 13 Department of Psychiatry and Behavioral Sciences, Johns Hopkins University, Baltimore, MD USA; 14 Divisions of Epidemiology and Biostatistics, Dalla Lana School of Public Health, Toronto, Canada; 15 Department of Statistical Sciences, Faculty of Arts and Science, Toronto, Canada; 16 Department of Psychiatry, Faculty of Medicine, University of Toronto, Toronto, Canada; 17 Waypoint Centre for Mental Health Care, Penetanguishene, ON Canada; 18 University of Milan, Milano, Italy; 19 Roy J. and Lucille A. Carver College of Medicine, University of Iowa, Iowa City, IA USA; 20 Yale Child Study Centre, New Haven, CT USA; 21 Faculdade de Medicina da Universidade de São Paulo (FMUSP), São Paulo, Brazil; 22 Instituto Nacional de Psiquiatría Ramón de la Fuente Muñiz, Mexico City, Mexico; 23 Icahn School of Medicine at Mount Sinai, New York, NY USA; 24 Utrecht University, Utretch, The Netherlands; 25 Ospedale San Raffaele Milano, Milano, Italy; 26 IRCSS Santa Lucia Foundation, Rome, Italy; 27 University of Southern California Keck School of Medicine, Los Angeles, CA USA; 28 University of Illinois at Chicago, Chicago, IL USA; 29 Yale University, New Haven, CT USA; 30 Bio4Dreams, Milano, Italy; 31 Vanderbit University, Nashville, TN USA; 32 AP-HP, Robert Debré Hospital, Paris, France; 33 Amsterdam UMC, location AMC, Amsterdam, The Netherlands; 34 QIMR Berghofer Medical Research Institute, Brisbane, Australia; 35 University of New South Wales, Sydney, Australia; 36 Ludwig-Maximilians-University Munich, Munich, Germany; 37 Columbia University Medical Centre, New York, NY USA; 38 Harvard Medical School, Boston, MA USA; 39 University Medicine Greifswald, Greifswald, Germany; 40 Alpert Medical School of Brown University, Providence, RI USA; 41 University Hospital of Psychiatry Zürich, University of Zürich, Zürich Centre for Integrative Human Physiology, Neuroscience Center Zürich, Zürich, Switzerland; 42 National Institute of Mental Health, Bethesda, MD USA; 43 Massachusetts General Hospital, Boston, MA USA; 44 McLean Hospital, Belmont, MA USA; 45 University of Chicago, Chicago, IL USA; 46 Centre for Addiction and Mental Health, Toronto, ON Canada; 47 Janssen Pharmaceuticals, Spring House, PA USA; 48 State University of New York, Downstate Health Sciences University, Brooklyn, NY USA; 49 Harvard T.H. Chan School of Public Health, Boston, MA USA; 50 Grupo Médico Carracci, Mexico City, Mexico; 51 Hôpitaux universitaires Henri-Mondor, Créteil, France; 52 Stellenbosch University, Stellenbosch, South Africa; 53 University of California, Irvine, Irvine, CA USA; 54 Johns Hopkins University, Baltimore, MD USA; 55 University of Florida College of Medicine, Gainseville, FL USA; 56 Aarhus University, Aarhus, Denmark; 57 Karolinska, Stockholm, Sweden; 58 Dalhousie University, Nova Scotia, NS Canada; 59 UCLA Semel Institute for Neuroscience and Human Behavior, Los Angeles, CA USA; 60 Instituto Nacional de Medicina Genómica (INMEGEN), Mexico City, Mexico; 61 VU University Amsterdam, Amsterdam, The Netherlands; 62 Sunnybrook Health Sciences Centre, Toronto, ON Canada; 63 Charité - Universitätsmedizin, Berlin, Germany; 64 University of Cologne, Cologne, Germany; 65 Federal University of Bahia, Salvador, Brazil; 66 Amsterdam University Medical Center, Amsterdam, The Netherlands; 67 SA MRC Unit on Risk & Resilience in Mental Disorders, University of Cape Town, Cape Town, South Africa; 68 University of British Columbia, BC Children’s Hospital Research; BC Mental Health and Substance Use Research, Vancouver, BC Canada; 69 New York State Psychiatric Institute, New York, NY USA; 70 University Hospital Bonn, Bonn, Germany; 71 Takeda Pharmaceuticals, Cambridge, MA USA

**Keywords:** Genomics, Human behaviour

## Abstract

Using a novel trait-based measure, we examined genetic variants associated with obsessive-compulsive (OC) traits and tested whether OC traits and obsessive-compulsive disorder (OCD) shared genetic risk. We conducted a genome-wide association analysis (GWAS) of OC traits using the Toronto Obsessive-Compulsive Scale (TOCS) in 5018 unrelated Caucasian children and adolescents from the community (Spit for Science sample). We tested the hypothesis that genetic variants associated with OC traits from the community would be associated with clinical OCD using a meta-analysis of all currently available OCD cases. Shared genetic risk was examined between OC traits and OCD in the respective samples using polygenic risk score and genetic correlation analyses. A locus tagged by rs7856850 in an intron of *PTPRD* (protein tyrosine phosphatase δ) was significantly associated with OC traits at the genome-wide significance level (*p* = 2.48 × 10^−8^). rs7856850 was also associated with OCD in a meta-analysis of OCD case/control genome-wide datasets (*p* = 0.0069). The direction of effect was the same as in the community sample. Polygenic risk scores from OC traits were significantly associated with OCD in case/control datasets and vice versa (*p*’s < 0.01). OC traits were highly, but not significantly, genetically correlated with OCD (*r*_g_ = 0.71, *p* = 0.062). We report the first validated genome-wide significant variant for OC traits in *PTPRD*, downstream of the most significant locus in a previous OCD GWAS. OC traits measured in the community sample shared genetic risk with OCD case/control status. Our results demonstrate the feasibility and power of using trait-based approaches in community samples for genetic discovery.

## Introduction

Obsessive-compulsive disorder (OCD) is a common (1–2% prevalence)^1^ psychiatric disorder characterized by intrusive, recurrent thoughts and repeated, ritualized behaviors. Up to 50% of OCD cases have a childhood-onset (before the age of 18)^2^, which is more heritable than adult-onset OCD^3^. Two genome-wide association studies (GWAS) in clinical samples with mixed ages of OCD-onset and a meta-analysis of these studies did not identify genome-wide significant loci^4–6^. The most significant loci from previous GWAS include SNPs within *DLGAP1*, *BTBD3, GRID2*, and one close to *PTPRD*. Using obsessive-compulsive (OC) symptoms rather than a clinical diagnosis, a study of adult twins identified a genome-wide significant SNP in *MEF2B* (rs8100480)^7^. However, this SNP was not replicated in an independent sample^5^.

**Fig. 1:**
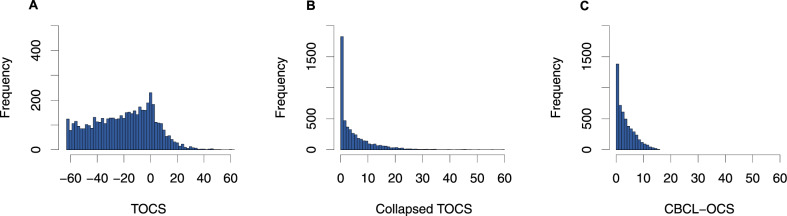
Distribution of OC trait measures. Histograms of **A** Toronto Obsessive-Compulsive Scale (TOCS) total score, **B** total score for collapsed TOCS items (all negative scores converted to zero for each item) and **C** the Child Behavior Checklist – Obsessive-Compulsive Scale (CBCL-OCS). *n* = 5018.

We conducted a GWAS of quantitative OC traits in a large pediatric, community-based sample: Spit for Science^8,9^. We measured OC traits using the Toronto Obsessive-Compulsive Scale (TOCS; https://lab.research.sickkids.ca/schachar/resources-and-tools/)^8^. This heritable measure^10^ includes negative scores that represent ‘strengths’ (e.g., never upset when their belongings are rearranged) and positive scores that represent ‘weaknesses’ (e.g., very upset when their belongings are rearranged). We reasoned that a strength-to-weakness format would generate scores with a more normal distribution in a community sample^8^ than those observed with typical OCD scales and would therefore boost the power of genetic discovery^11^. Typical OCD trait measures generate J-shaped distributions because their format calls for ratings of symptoms from absence to presence (score of zero to a positive integer). A j-shaped distribution is especially likely when using typical OCD measures in a community sample where the prevalence of OC symptoms is low and most people would get scores of zero^12^. This j-shaped distribution can be replicated with the TOCS by collapsing the ‘strengths’ (i.e., negative scores) into scores of zero (Fig. 1). We tested the hypothesis that the distribution of TOCS scores would boost the power of genetic discovery^11^ by running a GWAS with the collapsed TOCS measure as well as the full distribution. We characterized the genetic associations for TOCS by conducting gene-based analyses, examining brain expression quantitative trait loci (eQTLs) of the most significant loci, estimating SNP-based heritability and genetic correlations of total OC trait scores with other medical/mental health disorders and traits. We also examined if the most significant loci from the previous GWAS of OC symptoms^7^ replicated in our study. Finally, we tested the hypothesis that OC traits in the community share genetic risk with OCD by examining individual genetic variants, genetic correlations, and polygenic risk between OC traits in Spit for Science and three independent OCD case/control samples.

## Subjects and methods

### OC traits

#### Participants

The Spit for Science sample is described in detail elsewhere^9^. Briefly, the sample included 15,880 participants with complete demographic, questionnaire, and family information (mean age = 11.1 years [SD 2.8]; 49.4% female) from the 17,263 youth (6–18 years of age) recruited at the Ontario Science Centre over 16 months. Informed consent, and assent where applicable, were obtained using a protocol approved by the local Research Ethics Board at the Hospital for Sick Children. Participants provided a saliva sample in Oragene saliva kits (OG-500; DNA Genotek, Ottawa, Canada) for genetic analyses. See the supplement for details.

#### OC trait measure

We measured parent- and self-reported OC traits within the last 6 months using the TOCS, a 21-item questionnaire described previously^8,10^. Each item was scored on a 7-point Likert scale ranging from −3 (‘far less often than others of the same age’) to +3 (‘far more often than others of the same age’). A score of zero was designated as an average amount of time compared to same-age peers. The TOCS total score was standardized into a *z*-score to account for age, sex, and questionnaire respondent (parent or self). Details of *z*-score creation are described in the supplement. We tested the impact of the strength/weakness structure of the TOCS by re-scoring the TOCS to convert all negative scores for individual items to zero before summing scores (i.e., no scores less than 0, which collapsed the left side of the distribution). We also compared the TOCS to an additional OCD symptom measure with a j-shaped distribution: The Obsessive-Compulsive Scale of the Child Behavior Checklist (CBCL-OCS)^13^. Each of the eight CBCL-OCS items was scored on a scale of 0–2 (0 = not true; 1 = somewhat/sometimes true; and 2 = very/often true) and was summed to generate a total score (range: 0–16). This ‘collapsed’ TOCS total score, with a cluster of scores at zero, created a distribution similar to the CBCL-OCS (Fig. 1).

#### Genetic data

DNA was extracted manually from saliva using standard methods (see the supplement for additional details). We excluded any samples with concentrations <60 ng/µl and insufficient quality based on agarose gels. We genotyped 5645 samples on the Illumina HumanCoreExome-12v1.0_B (HumanCore) and 192 samples on the Illumina HumanOmni1-Quad V1.0_B (Omni) bead chip arrays (Illumina, San Diego, CA, USA) at The Centre for Applied Genomics (Hospital for Sick Children, Toronto, CA). There were 538,448 markers on the HumanCore and 1,140,419 markers on the Omni array.

Quality control (QC) was conducted separately for each array using standard methods with PLINK v1.90^14^. Sample exclusion and selection criteria are described in the supplemental methods and Supplemental Figure S1. Imputation was performed separately for all platforms and sample sets, using Beagle v4.1 using the data from phase 3, version 5 of the 1000 Genomes project for reference (http://bochet.gcc.biostat.washington.edu/beagle/1000_Genomes_phase3_v5a/). We excluded individuals who were non-Caucasian based on principal component (PC) analysis and included only one participant from each family (inferred sibs or half-sibs, see supplement and Supplemental Figure S2). Genetic data will be available through the SickKids Healthy Kids Biobank.

#### Analyses

GWAS was conducted using R (v3.5.1). Our primary analysis tested if imputed dosage and standardized TOCS total score were associated using a linear regression model that included the top three PCs and genotyping array as covariates. We included SNPs with a minor allele frequency (MAF) > 1%, allelic *R*^*2*^ imputation quality (AR2 > 0.6) and used the standard genome-wide threshold of *p* ≤ 5 × 10^−8^. We also tested if any genome-wide significant variants from the analysis with the standardized scores were still significant using a non-standardized TOCS score. For these analyses, age, sex, respondent, and their 2- and 3-way interactions were used as covariates in addition to the above (interactions were included to mimic the construction of the Z scores, which were calculated independently in age-, sex- and respondent-defined bins; see supplement).

In secondary analyses, we evaluated the association between SNPs and the collapsed TOCS score, and between SNPs and CBCL-OCS, using zero-inflated negative binomial likelihood ratio tests, using the function *zeroinfl* from the *R* package *pscl* (v1.5.2). This model was chosen because of the high proportion of zero scores that created a j-shaped distribution. The test is a mixture of two models: a negative binomial model, which contributes to zero and positive scores, and a logit model, which contributes to possible inflation of zero scores (point mass at 0) compared to what a negative binomial model predicts. These analyses used non-standardized scores for the collapsed TOCS and CBCL-OCS so the model adjusted for the covariates and the association of SNP allele dosage with the OC trait scores is tested against the null of having no effects on both the logit part and the negative binomial part using likelihood ratio tests.

We subsequently used FUMA to conduct a gene-based GWAS of the TOCS standardized total score with MAGMA using a Bonferonni correction for the number of protein-coding genes included^15^ (fuma.ctglab.nl).

We tested each genome-wide significant variant for co-localization with brain eQTLs using LocusFocus^16^ (https://locusfocus.research.sickkids.ca/). We examined the 14 GTEx sets from brain tissue types and examined SNPs within ±1 Mbp of each SNP.

We estimated SNP heritability using both GCTA^17^ v1.91.2-beta (http://cnsgenomics.com/software/gcta/) with further exclusion of cousins and SNPs with AR2 > 0.9 and LDSC^18^ (v1.0.0, https://github.com/bulik/ldsc) calculated from SNPs in HapMap3. We used LDSC^19^ to examine the genetic correlation of TOCS total scores with the 850 phenotypes available on LD Hub (http://ldsc.broadinstitute.org/ldhub/).

Finally, we examined the *p*-values and effect sizes of the top variants from the present study in the only previous GWAS of OC symptoms^7^ of 6931 twins and sibs from the Netherlands Twin Registry (only 20 loci reported in the results from the previous paper were also in SNP set from the present study).

### OCD case/control

#### Participants

For validation analyses, we investigated three independent OCD case/control cohorts: (1) the International OCD Foundation Collaborative (IOCDF-GC) and OCD Collaborative Genetics Association Studies (OCGAS) meta-analysis^6^, (2) the Philadelphia Neurodevelopmental Cohort (PNC) from the Children’s Hospital of Philadelphia (CHOP)^20^, and (3) the Michigan/Toronto OCD Imaging Genomics Study^21^. See the supplement and Table 1 for sample sizes.

**Table 1:** Overview of samples.

OC trait sample	Phenotype	Age	Total samples	Cases	Controls
Spit for Science	TOCS, CBCL-OCS	6–18 years	5018	n/a^a^	n/a
All replication	Phenotype	Age of onset			
IOCDF/OCGAS	Clinician-diagnosed (DSM-IV)	Child and adult	9725	2688	7037
CHOP	Case/control status based on GO-ASSESS symptoms	<18 years of age	1775	406	1369
Michigan/Toronto	Clinician-diagnosed (DSM-IV)	<18 years of age	480	275	205
Total meta-analysis			11,980	3369	8611

^a^There were 62 individuals that had a parent- or self-reported diagnosis of OCD. Number of samples reflects samples included in analyses (i.e., after quality control analyses). IOCDF/OCGAS = International OCD Foundation Collaborative and OCD Collaborative Genetics Association Studies meta-analysis sample, CHOP = the Philadelphia Neurodevelopmental Cohort (PNC) from the Children’s Hospital of Philadelphia, TOCS = Toronto Obsessive-Compulsive Scale, CBCL-OCS = Child Behavior Checklist – Obsessive-Compulsive Scale, DSM-IV = Diagnostic and Statistical Manual of Mental Disorders, 4th edition.

#### Analyses

To validate findings from the GWAS of the TOCS total score, we combined the GWAS summary statistics from each OCD cohort using a fixed-effect inverse variance meta-analysis. For completeness, we also conducted a meta-analysis of the summary statistics from the GWAS of the TOCS total score with the OCD samples using a modified sample size-based weighted meta-analysis method for combining continuous and categorical variables^22^ (see supplemental methods for details). In brief, this approach weights the two sets of results based on their SNP heritability and genetic correlation. Polygenic risk score (PRS) analyses were performed using LDpred v1.06^23^ (see supplement). First, we derived PRS for TOCS from the Spit for Science sample and tested their association with case/control status in the combined OCD cohorts (target sample: CHOP, Michigan/Toronto and a subset of the IOCDF-GC/OCGAS—see supplement). Second, we derived PRS from the combined OCD cohorts and tested their association with the standardized TOCS total score in the Spit for Science sample (target sample). We examined the potential shared genetic risk between the Spit for Science and the meta-analyzed OCD samples using genetic correlations estimated with LDSC^19^.

## Results

### OC traits

**Fig. 2:**
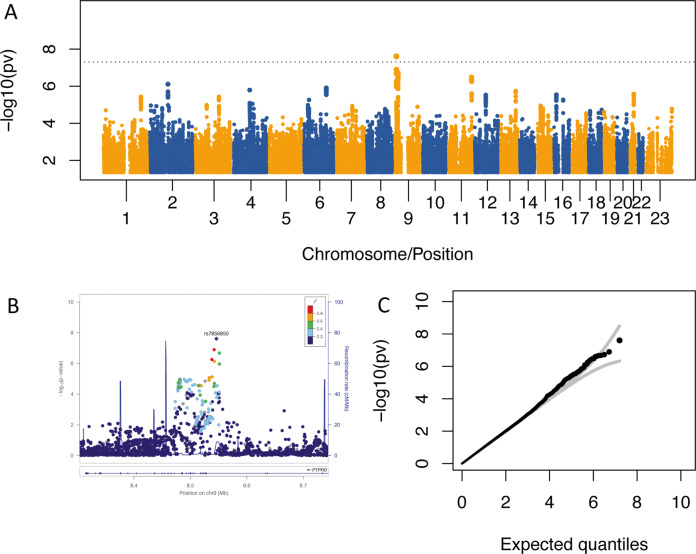
Genome-wide significant locus in *PTPRD* associated with OC traits in Spit for Science. **A** Manhattan plot for GWAS of the TOCS standardized total score. rs7856850 in one of the introns of *PTPRD* surpassed the genome-wide threshold (*p* = 5 × 10^−8^; gray line). **B** Locus zoom plot for the genome-wide significant locus from the GWAS of the TOCS standardized total score. **C** QQ Plot for the GWAS of the TOCS standardized total score. *n* = 5018.

We used 5018 participants for GWAS analyses after sample exclusion and selection (see supplement and Supplemental Figures S1/S2). In the primary analysis, rs7856850 in *PTPRD* was significantly associated with TOCS total scores at the genome-wide level (*p* = 2.48 × 10^−8^, *β* = 0.14, s.e. = 0.025, *R*^*2*^ = 0.618%: Fig. 2A, most significant loci listed in Supplemental Table S1). Several variants in this region that approached genome-wide significance were in linkage disequilibrium (LD) with rs7856850, which was genotyped on both the HumanCore and OMNI arrays (Fig. 2B). The inflation factor *λ* was 1.008 while the intercept of LD score regression was 1.003 and not significantly different from 1 (s.e. = 0.007, *p* = 0.66; Fig. 2C). rs7856850 was still associated with TOCS total scores using raw instead of standardized scores (*p* = 2.75 × 10^−8^, *R*^*2*^ = 0.615%; data not shown). There was no eQTL data for the SNP in *PTPRD*, rs7856850, in LocusFocus^16^, or in the most recent version of GTEX v8^24^.

When we analyzed the collapsed TOCS total score and the CBCL-OCS, the genome-wide significant locus for the TOCS total score rs7856850 was no longer genome-wide significant, although the remaining effect was in the same direction and had the same direction of effect (*p* = 0.00045 and *p* = 0.025, respectively; see supplement for details). For both collapsed TOCS and the CBCL-OCS, the A allele was associated with both higher scores (collapsed TOCS: *β* = 0.0735, s.e. = 0.0292, CBCL-OCS: *β* = 0.0465, s.e. = 0.020) and lower proportion of zero scores (collapsed TOCS: *β* = −0.231, s.e. = 0.104, CBCL-OCS: *β* = −0.126, s.e. = 0.179).

A gene-based GWAS of the TOCS standardized total score using MAGMA on the FUMA platform did not identify any genome-wide significant genes (at a Bonferroni-corrected level *p* = 0.05/19369 protein-coding genes = 2.58 × 10^−6^). The most significant genes were *SH3GL2* (*p* = 4.21 × 10^−6^, *z* = 4.45); *RRN3* (*p* = 6.23 × 10^−6^, *z* = 4.37), and *PDXDC1* (*p* = 1.10 × 10^−5^, *z* = 4.24; Supplemental Figure S3). *PDXDC1* and *RRN3* have overlapping coding regions.

The heritability of the TOCS total score was *h*^*2*^ = 0.068 (s.e. = 0.052, *p* = 0.19) using GCTA and *h*^*2*^ = 0.071 (s.e. = 0.060; *p* = 0.24) using LDSC when the intercept was constrained to 1. TOCS total score was not significantly associated with any phenotypes on LD Hub (see supplement).

One of the top-ranked SNPs from a previous GWAS of OC symptoms^7^ was nominally associated with TOCS total scores in the Spit for Science sample with the same direction of effect (rs60588302, *p* = 0.025). This SNP is in the same region as our most significant locus (9p24.1), but not in LD (*r*^*2*^ = 0.004, *D*′ = 0.517). Another 16 of the reported most significant loci in den Braber^7^, including a variant in *MEF2BNB* (rs8100480) that was genome-wide significant in their sample, had effects in the same direction but were not significantly associated in the current sample (Supplemental Table S2).

### OCD case/control

**Fig. 3:**
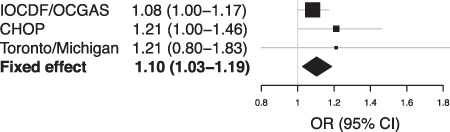
Validation of locus in PTPRD in OCD samples. Forrest plot of genome-wide significant variant (rs7856850) across all the replication samples and sub-samples: (1) IOCDF/OCGAS (International Obsessive-Compulsive Disorder Foundation Collaborative and OCD Collaborative Genetics Association Studies samples), (2) CHOP (Philadelphia Neurodevelopmental Cohort (PNC) from the Children’s Hospital of Philadelphia, and (3) Michigan/Toronto OCD Imaging Genomics Study. Total cases: 3369; total controls: 8611. OR = odds ratio.

Following standard QC and sample exclusion where applicable (see supplement), we had a total of 3369 cases and 8611 controls in our validation samples (Table 1). We tested if the genome-wide SNP associated with TOCS total scores in Spit for Science were also associated with OCD in the meta-analysis of case/control cohorts. rs7856850 was associated with increased odds of being an OCD case (*p* = 0.0069, OR = 1.104 per A allele [95% confidence limit 1.03–1.19], Fig. 3, Supplemental Figure S4). When the summary statistics of the TOCS total score were meta-analyzed with the OCD cohorts, there were no genome-wide significant variants (Supplemental Figure 5). rs7856850 approached genome-wide significance *p* = 1.2 × 10^−7^ when using conventional sample size-weighted meta-analysis but fell to *p* = 0.00054 when the sample sizes were adjusted for SNP heritability and genetic correlation (see supplemental results for details).

**Fig. 4:**
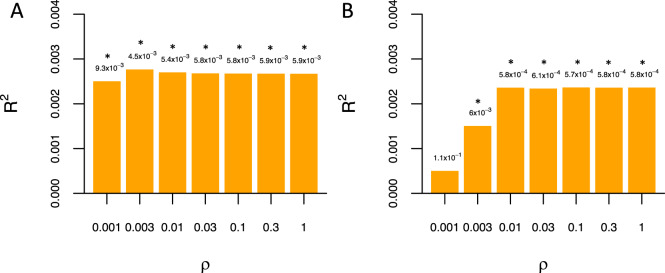
OC traits in the community and OCD share polygenic risk. **A** Variance explained (*R*^2^) in OCD case/control status in replication samples by polygenic risk for OC traits from Spit for Science **B** variance explained in OC traits in Spit for Science sample by polygenic risk for OCD from replication samples across a range of prior proportion of causal variants (*ρ*). Analyses conducted using LDpred. *p < 0.01.

The genetic correlation between standardized TOCS total scores and OCD meta-analysis was *r*_g_ = 0.71 (s.e. = 0.382; *p* = 0.062; 95% CI: [−0.04,1]) when intercepts are constrained to 1. Figure 4A shows that PRS calculated for TOCS total scores was significantly associated with increased odds of being a case in the meta-analyzed OCD samples (Nagelkerke’s pseudo *R*^*2*^ = 0.277%, *p* = 0.0045 at *ρ* = 0.003). Figure 4B shows that PRS constructed from the OCD sample were significantly associated with TOCS total scores in Spit for Science (*R*^*2*^ = 0.24%; *p* = 0.00057 at *ρ* = 0.1).

## Discussion

Using a trait-based approach in a community sample, we identified a genome-wide significant variant associated with OC traits (rs7856850) that was also associated with OCD case/control status. Polygenic risk and genetic correlation findings showed sharing of genetic risks between OC traits in the community and OCD case/control status in independent samples.

The genome-wide significant variant (rs7856850) associated with OC traits is in an intron of the consensus transcript of *PTPRD* that codes for protein tyrosine phosphatase δ. No eQTLs have been calculated yet for rs7856850 (GTEx V8)^24^. To validate this finding, we tested if this SNP was also associated with OCD in a meta-analysis of three independent cohorts. The significant association of rs7856850 with OCD case/control makes it the first variant associated with OC traits and OCD. For completeness, we presented genome-wide results for the meta-analysis of the OCD cohorts as well as a meta-analysis of TOCS total score with the OCD cohorts, where no genome-wide significant findings were revealed. However, the direction of effect for rs7856850 was in the same direction in all samples. The small size of the OCD cohorts likely precluded finding genome-wide significant SNPs. In the meta-analysis of OC traits and OCD, summary statistics were combined using sample size-based weights that were modified and calibrated to account for SNP heritability to reflect differences in power and ascertainment between continuous (OC traits) and categorical (OCD case/control) designs^25^. The low SNP heritability of TOCS severely down-weighted the OC trait sample size while up-weighting the already underpowered OCD case/control cohorts. Larger samples will be helpful to confirm the results from the present study.

Previous GWAS of OCD symptoms or diagnosis identified variants that approached significance in the region around *PTPRD*. However, those variants were independent of the locus found in our study^4,7^. These observations support a possible role of the 9p24.1 region in OCD. The 9p region is also the location of one of the strongest linkage peaks in earlier genome-wide linkage studies of pediatric OCD^26,27^. Rare CNVs in *PTPRD* have been identified in cases with OCD^21^ and ADHD^28^. SNPs in *PTPRD* were genome-wide significantly associated with ASD^29^, restless legs syndrome^30^, and self-reported mood instability^31^. *Ptprd*-deficient mice show learning deficits and altered long-term potentiation magnitudes in hippocampal synapses^32^. *PTPRD* is expressed highly in the brain compared to non-brain tissues, especially in myelinating axons and growth cones^33,34^ in the prenatal cerebellum^35^. The presynaptically located PTPRD is involved in axon outgrowth and guidance^36^ and interacts with postsynaptic proteins such as Slitrk-2, interleukin-1 receptor, and TrK to mediate synapse adhesion and organization in mice^37,38^ and the development of excitatory and inhibitory synapses^39^. Members of the Slitrk and interleukin protein families have been associated with OC behaviors in humans and mice^40,41^.

Our results show that OC traits in the community share genetic risk with OCD. Polygenic risk for OC traits was associated with OCD case/control status and vice versa. OC traits and OCD case/control status also were substantially, but not significantly, genetically correlated. This estimate is higher than reported in a recent study (*r*_g_ = 0.42, *p* = 0.095; 50)^43^. Lack of power is the most likely explanation for the absence of a significant result. Previous studies of other psychiatric disorders reported shared genetic risk between traits and diagnoses, with polygenic risk and genetic correlations similar to what we report for OC traits and OCD case/control status^25,31,44^. The shared genetic risk between OC traits and OCD supports the hypothesis that an OCD diagnosis could represent the high extreme of OC traits that are widely distributed in the general population. One implication of this finding is that population-based samples with quantitative trait measures can serve as a powerful complementary approach to case/control studies to accelerate gene discovery in psychiatric genetics.

SNP-based heritability for OC traits in the current sample was not significant in line with previous studies. Previous research reports lower SNP-based heritability for self-reported OC symptoms (0.058)^42^ than for clinical OCD (0.28–0.37)^6,45^. A similar trend for lower SNP heritability in traits vs. diagnosis has been observed for ADHD^25,46^. The reason for the disparity in SNP heritability between traits and diagnosis is unclear as there are several differences that may play a role including informant (parent/self vs. teacher or clinician)^46^, type of measurement (categorical vs. quantitative), consideration of impairment, and timing (cross-sectional vs. lifetime symptoms). Regardless of a non-significant SNP heritability for OC traits from our sample, we still identified and validated a genome-wide significant variant.

The TOCS scale is similar to existing OC trait/symptom measures in item content but is unlike existing scales in that it measures OC traits from ‘strengths’ to ‘weaknesses’. As a result, the distribution of the total score is closer to a normal distribution than the j-shaped distributions typically observed with most symptom-based scales that rate behaviors from zero to a positive integer^10^ (e.g., not at all to quite a lot). Our results indicate that the distribution of the OC trait measure impacts power to identify genome-wide significant associations. A ‘strengths’ to ‘weaknesses’ measure identified a genome-wide significant association. However, when we collapsed the ‘strength’ end of the TOCS distribution to zero, the significance of this variant was substantially reduced to below genome-wide significance, although the effect was in the same direction. The same effect was observed using another OC measure that generates a j-shaped distribution: CBCL-OCS. One implication of our results is that there is genetic information in the ‘strengths’ end of the distribution captured by the TOCS. This information would be lost in scales that only measure ‘weaknesses’, particularly in community samples where the prevalence of clinically significant OC symptoms is relatively low. Trait-based scales that capture ‘strengths’ and ‘weaknesses’ and have a less skewed distribution could improve power to identify genome-wide hits and variants associated with disorders, especially in population samples.

The results of this study should be considered in light of its limitations. Although our sample was large enough to detect a genome-wide significant locus that was also significant in meta-analyzed OCD case/control cohorts, substantially larger samples will be needed to identify most of the contributing common variants. The current version of the TOCS measures OC traits cross-sectionally, which does not account for symptom waxing and waning and does not measure impairment directly. However, our polygenic risk and genetic correlation analyses show that OC traits and OCD share genetic risk, suggesting that the TOCS is capturing traits that are likely to be on a continuum with OCD.

## Conclusions

We identified the first genome-wide significant variant for OC traits that was also associated with OCD case status. Power to detect a genome-wide association was impacted by the distribution of the OC trait measure. OC traits and OCD share genetic risks supporting the hypothesis that OCD represents the extreme end of widely distributed OC traits in the population. Trait-based approaches in community samples using measures that capture the whole distribution of traits is a powerful and rapid complement to case/control GWAS designs to help drive genetic discovery in psychiatry.

## Supplementary information

Supplemental Methods and Results
