# Supplementary material for: Genome-wide association study of pediatric obsessive-compulsive traits: shared genetic risk between traits and disorder
Source: Transl Psychiatry. 2021 Feb 2;11:91. doi: 10.1038/s41398-020-01121-9 (PMC7870035; doi:10.1038/s41398-020-01121-9)
Supplement: Supplementary file 1 — Supplemental Methods and Results [file 41398_2020_1121_MOESM1_ESM.pdf]

Supplemental Methods and Materials

OC Traits

Participants

We collected behavioural information about the participants from themselves if they were deemed capable of self-reporting (18.6%; typically 12 years of age or older) or from their parents (81.4%). Ethnicity was estimated using a self-report questionnaire and confirmed using genetic data (see below). We collected information about whether the participant had ever received a diagnosis of, or had been treated, for OCD. The sample was highly enriched for siblings with 51.6% of the sample (n = 8190) having at least one sibling who also participated in the study (total number of families = 3816).

Z-Score Estimation

We created standardized z-scores that accounted for age, sex, and respondent-type (parent or self), which were each associated with the TOCS total score ($p < 0.05$). In order to eliminate ties when estimating z-scores, the modified total scores were modeled separately by respondent, with age and sex as covariates, treating family as a random effect, and residual scores were used to calculate the z-scores. Children and youth were divided into thirty groups according to respondent, gender and age. For parent respondents, groups included every integer year of age from 6-15. For self-respondents, integer year age groups were created for ages 13-17. A normally distributed quantitative score was assigned to each subject by sorting the residuals and substituting a z-score corresponding to their empirical percentile within each group. Z-scores were assigned within each of the thirty groups separately so that the distribution of scores would be comparable across age, gender, and respondent.

Preparation of Genetic Samples & Genotyping

To precipitate any possible carbohydrates in the sample, we centrifuged the samples for an additional 10 min at 10 000 RPM and removed any formed pellet from the sample. DNA was quantified using the

Quanti-iT, Pico Green® dsDNA kit from Invitrogen (Thermo Fisher Scientific) and samples with concentrations < 60ng/µl were excluded (6.5% of all extracted cases). DNA was subsequently aliquoted and stored at -80°C. Prior to conducting the microarrays, DNA quality was verified using agarose gels and 98.5% of samples had sufficient DNA quality.

On each 96 well plate, we also genotyped an individual from a Caucasian HapMap trio (CEPH) as a quality control[1] with each sample genotyped approximately 20 times. Genotypes were called using GenomeStudio (Illumina, San Diego, CA, USA) separately for the HumanCoreExome (GenomeStudio v 1.9.4) and HumanOmni1 samples (GenomeStudio v2009.2).

Genotyping QC and Selection of Participants for Genetic Analysis

SNP position and annotation information were based on NCBI36 for Omni and on Genome Reference Consortium 37 (GRCh37) for HumanCore. Samples were excluded if their call rate was below 97%, a heterozygosity rate of 6 times the interquartile range from the closest quartile and/or their predicted and reported sex were mismatched. SNPs were excluded if they had call rates below 97%, they deviated from the rules of Hardy-Weinberg equilibrium at an FDR <1% (based on a set of homogeneous samples in terms of ancestry) and/or were duplicates of other SNPs, based on position and alleles (only the SNP with the highest call rate was retained). Nine participants were successfully genotyped on both platforms and we only kept data from the HumanCoreExome array. Samples were also excluded from statistical analysis if they did not have four grandparents of reported Caucasian descent, had a sex aneuploidy based on copy number analysis, did not have a standardized TOCS total score or had a parent- or self- reported diagnosis of autism spectrum disorder (ASD). Participants with ASD were excluded because the TOCS queries some behaviours that are common in ASD and we wanted to reduce the chance of phenocopies in our sample[2]. Concordance of the HapMap trio samples genotyped on each HumanCore plate, were also >99.99%.

Imputation

A/T and C/G genotyped SNPs were removed prior to imputation. Allele coding on the X chromosome were coded as 0,1,2 for females and 0,2 for males.

Ethnicity and Principal component calculation

Figure S1 outlines the number of samples removed during QC. First, we excluded participants that did not have four grandparents of reported Caucasian descent. Next, principal components (PCs) were calculated from a set of autosomal, bi-allelic ancestry informative markers (AIM), calculated from samples from phase 3 of the 1000 Genomes project. We first pruned SNPs for linkage disequilibrium (r^2<0.2 in 1500 kbp windows). Then, for each continental population, the top 1% SNPs with the largest frequency differences between that population and all others were retained. We ignored SNPs in the intervals chr8:7000000-13000000 [hg19] (8p23 inversion) and chr6:25000000-34000000 (MHC).

Participants' AIMs were extracted from the imputed data sets, as long as their imputation quality was AR2>0.8. Hard genotype calls were used. To identify outliers with respect to ancestry, data from participants were combined with samples from the 1000 Genomes project. PCs were calculated using plink v1.90, and we excluded outliers in any of the first 3 PCs calculated from ancestry informative markers and from combining the participants with samples from phase 3 of the 1000 genomes project (see below; see Figure S2). Once ancestry outliers were removed, we recomputed PCs without 1000 Genomes samples.

Relatedness

The set of AIM SNPs (average observed heterozygosity of 0.41) was used to assess relatedness among the participants, using the "genome" option of plink. The estimated proportion π of autosomal genome identical by descent was inspected in pairs of participants. Networks of related participants with

estimated pairwise $\pi > 0.18$ (inferred half-sibs or closer) was constructed. For Spit for Science samples, one participant from each network was retained for GWAS analysis, based on the highest standardized TOCS value. For the case-control studies, selection was based on participants being a case and/or older.

OCD Case/Control

Participants

OCD Cohort 1: Meta-Analysis of the International Obsessive-Compulsive Disorder Foundation Collaborative (IOCDF-GC) and OCD Collaborative Genetics Association Studies (OCGAS) Samples. This meta-analysis of two published GWASs of OCD in European Caucasians is described in detail elsewhere[3]. The study consisted of 2688 patients with OCD based on DSM-IV criteria and 7037 genomically matched controls. Summary statistics from the IOCDF-GC/OCGAS GWAS were downloaded from https://www.med.unc.edu/pgc/results-and-downloads/ocd/ (file ocd_aug2017.gz). Individual level data was accessed through dbGaP accession number phs000092.v1.p.

OCD Cohort 2: Philadelphia Neurodevelopmental Cohort (PNC) from the Children's Hospital of Philadelphia (CHOP). The PNC sample is described in detail elsewhere[4] and the data were obtained under approval from dbGaP phs00607.v2.p2. Briefly, the PNC sample is comprised of 9428 participants aged 8-21 years recruited from over 50 000 children genotyped from a blood sample by the Center of Applied Genomics after coming to CHOP or a CHOP-affiliated clinic for pediatric care. Participants were recruited randomly after the sample was stratified by age, sex and ethnicity[5]. Participants completed a computerized structured screener based on the Kiddie-Schedule for Affective Disorders and Schizophrenia (K-SADS) called GO-ASSESS[6,7]. The GO-ASSESS section related to OCD asked about the lifetime presence of any obsessive or compulsive symptoms as well as the severity, level of impairment and age of onset of symptoms.

In our validation analyses, we included individuals genotyped on the Illumina Human610-Quadv1_B BeadChip array who self-reported as European Caucasian. The samples released were all previously

genotyped by the Center for Applied Genomics at The Children's Hospital of Philadelphia[8]. QC was conducted as described for the Spit for Science sample. Participants were categorized into two groups: 1) participants with at least one OC symptom and reported impairment from the symptom(s) were considered cases (*n* = 421), and 2) participants with no OC symptoms and reported impairment were considered controls (*n* = 1441). We tested the association between imputed genotypes and case/control status using logistic regression controlling for age, sex and three PCs.

OCD Cohort 3: Michigan/Toronto OCD Imaging Genomics Study. Children and their parents were recruited from four academic child psychiatry sites: The Hospital for Sick Children, McMaster University, University of Michigan, and Wayne State University. Recruitment and diagnosis procedures have been described in detail elsewhere[9]. All enrolled individuals had symptoms first identified before age 18. Informed consent or assent where applicable were obtained as approved by the respective institutional ethics review boards. The site clinical investigator — a child and adolescent psychiatrist — made lifetime and current axis 1 diagnoses using all sources of information according to DSM-IV criteria. OCD clinic samples (*n* = 353) and controls (*n* = 317) were genotyped on a variety of genotyping arrays: HumanCoreExome, PsychArray and Omni2.5. Each genotyping array was processed separately, using the same pipeline as for the Spit for Science samples. Only cases and controls were retained. Imputed data from all arrays were combined, and the association between imputed dosage and case-control status was assessed using logistic regression, using as covariates the first 3 PCs, age, sex and array identifier.

Analyses

Meta-Analysis

The summary statistics for the TOCS total score and OCD case/control are on different scales (quantitative and logistic respectively). These two sets of results were thus combined in a sample size-weighted meta-analysis by following the framework of Demontis[10], which accounts for the differences in scale by calculating an effective sample size for each study that incorporates the original sample sizes,

the heritability and genetic correlation of the traits, as well as the prevalence of OCD (here taken to be 0.02[11]).

Polygenic Risk Score Prediction

Data from IOCDF-GC/OCGAS that were used for PRS association analyses consisted of IOCDF-GC Ashkenazi Jewish (91 cases, 255 controls), IOCDF-GC European (1032 cases, 4100 controls), IOCDF-GC South African (98 cases, 157 controls) and OCGAS case/control dataset (344 cases, 1033 controls). For IOCDF-GC and OCGAS studies, the association analyses between PRS and case-control status were assessed with a logistic model, adjusted for sex and 20 ancestry dimensions from multidimensional scaling (as provided with the data); for CHOP and Michigan/Toronto, analyses were adjusted for the covariates listed above. When all studies were combined in a single PRS analysis, an additional indicator of the study was used as a covariate, and the logistic model included an interaction term between the study and the PRS to account for the between-study heterogeneity. The significance of the PRS was assessed by comparing an analysis of variance (ANOVA) of the full logistic model (with PRS and its interaction with study) to the nested model without PRS and its interaction. We used LDpred[12] to conduct the PRS calculations, which estimates a posterior mean effect size for each marker by using a Gaussian prior with point mass at zero (based on an unknown parameter ρ representing the fraction of causal markers) for the effect sizes and linkage disequilibrium (LD) information. As recommended, PRS were evaluated at the default ρ values of 1, 0.3, 0.1, 0.03, 0.01, 0.003 and 0.001, restricting to SNPs with imputation quality AR2>0.90, MAF>1% and present in HapMap3. We tested the association between either 1) TOCS total scores with polygenic risk for OCD case/control status at each ρ value, and 2) OCD case/control status with polygenic risk scores for TOCS scores at each ρ value. In all polygenic risk score analyses, we included genotyping array as well as PCs as covariates.

Supplemental Results

<u>OC Traits</u>

After sample exclusion and selection, 5018 participants were included in the GWAS analysis (out of 5645 genotyped on HumanCore and 192 genotyped on Omni. See Figure S1 for the number of samples removed during each step of QC.

The zero-inflated negative binomial distribution model was a good fit for the collapsed score (Cramer-von Mises goodness-of-fit p=0.83, compared to p=0.001 for a non-inflated negative binomial distribution, using estimated parameters).

Genetic Correlation with Other Mental Health/Medical Traits

On LD Hub, the positive correlation between TOCS total score and childhood IQ had the smallest p-value, however this correlation was not statistically significant (r_g=0.64; p=0.19, s.e.=0.48).

OCD Case/Control

CHOP

After sample exclusion/selection, 406 cases and 1369 controls remained for analysis, out of a total of 421 cases and 1441 controls that were genotyped. We excluded samples because of technical quality control (n = 11), non-European ancestry (n = 24) and relatedness to another participant (n = 52).

Michigan/Toronto OCD Imaging Genomics Study

A total of 690 DNA samples were genotyped on one or more arrays (HumanCoreExome n = 45, PsychArray n = 363, Omni2.5 n = 282). Forty-nine samples were removed after technical exclusion; two duplicated DNAs with non-matching genome were removed; 95 samples related to or duplicates of another sample were removed; 58 samples were removed due to ancestry; and six samples were excluded due to changes in consent. After sample exclusion and sample selection, 275 cases and 205 controls remained.

Meta-Analysis

Results of the genome-wide meta-analysis of TOCS total score and OCD case/control are presented in Supplementary Figure S5. There were no genome-wide significant results observed. In particular, significance of rs7856850 dropped to p=0.00054. This is partly explained by the smaller heritability for TOCS compared to OCD and the moderate genetic correlation, which severely downweighed the effective sample size of TOCS from 5018 to only 301, while upweighting the effective sample size of OCD from 12 067 to 14 859. Instead of effective sample sizes, using actual sample sizes as weight in the meta-analysis, the significance of rs7856850 is $p=1.2\times10^{-7}$ and no genome-wide significant results were observed (not shown). SNP heritability calculated with LDSC from the OCD meta-analysis was $h^2=0.21$ (s.e.=0.024; $p=7\times10^{-18}$).

Polygenic Risk Scores

For analyses that required individual level data from the Psychiatric Genomics Consortium (PGC), not all the data were available because of ethics approvals (IOCDFGC Dutch) and use of cases no longer included in the PGC sample (pseudo-case controls - OCGAS Trios).

References

1 International HapMap Consortium *et al.* A second generation human haplotype map of over 3.1 million SNPs. *Nature* 2007; **449**: 851–61.

2 Jiujias M, Kelley E, Hall L. Restricted, Repetitive Behaviors in Autism Spectrum Disorder and Obsessive–Compulsive Disorder: A Comparative Review. *Child Psychiatry Hum Dev* 2017; **48**: 944–959.

3 International Obsessive Compulsive Disorder Foundation Genetics Collaborative (IOCDF-GC) and OCD Collaborative Genetics Association Studies (OCGAS). Revealing the complex genetic

architecture of obsessive-compulsive disorder using meta-analysis. *Mol Psychiatry* 2018; **23**: 1181–1188.

4 Satterthwaite TD *et al.* NeuroImage The Philadelphia Neurodevelopmental Cohort : A publicly available resource for the study of normal and abnormal brain development in youth. *Neuroimage* 2015; **124**: 1115–19.

5 Satterthwaite TD *et al.* Neuroimaging of the Philadelphia Neurodevelopmental Cohort. *Neuroimage* 2014; **86**: 544–553.

6 Calkins ME *et al.* The Philadelphia Neurodevelopmental Cohort : constructing a deep phenotyping collaborative. *J Child Psychol Psychiatry* 2015; **56**: 1356–1369.

7 Calkins ME *et al.* The psychosis spectrum in a young U.S. community sample: findings from the Philadelphia Neurodevelopmental Cohort. *World Psychiatry* 2014; **13**: 296–305.

8 Glessner JT *et al.* Strong synaptic transmission impact by copy number variations in schizophrenia. *Proc Natl Acad Sci U S A* 2010; **107**: 10584–9.

9 Gazzellone MJ *et al.* Uncovering obsessive-compulsive disorder risk genes in a pediatric cohort by high-resolution analysis of copy number variation. *J Neurodev Disord* 2016; **8**. doi:10.1186/s11689-016-9170-9.

10 Demontis D *et al.* Discovery of the first genome-wide significant risk loci for ADHD. 2017; : 145581.

11 Boileau B. A review of obsessive-compulsive disorder in children and adolescents. Dialogues Clin. Neurosci. 2011; **13**: 401–411.

12 Vilhjálmsson BJ *et al.* MoVilhjálmsson, B. J., Yang, J., Finucane, H. K., Gusev, A., Lindström, S.,

Ripke, S., … Price, A. L. (2015). Modeling linkage disequilibrium increases accuracy of polygenic risk scores. The American Journal of Human Genetics, 97(4), 576–592. https://doi. *Am J Hum Genet* 2015; **97**: 576–592.

Figure Legends

Supplemental Figure S1: Flow Chart of Sample and SNP Exclusion for Spit for Science

Numbers reported within square brackets are overlapping within each step. Reported non-Caucasian = not all four grandparents were parent- or self-reported to be of Caucasian European descent. HCE = HumanCoreExome array, OMNI = OMNI1 array, TOCS = Toronto Obsessive-Compulsive Scale, MAF = minor allele frequency, ASD = autism spectrum disorder, PCA = principal component analysis for population stratification, AR2 = allelic R^2.

Supplemental Figure S2: Principal Component Analysis Plots for Population Stratification

Principal component (PC) analysis plots for the first three PCs showing outliers (light grey with circle with X) removed because they did not cluster with European samples (EUR, light blue). HCE = HumanCoreExome chip, omni = OMNI1 chip, AFR = African, AMR = Ad Mixed American, EAS = East Asian and SAS = South Asian.

Supplemental Figure S3: Gene-Based Genome-Wide Analysis of OC Traits in Spit for Science

Manhattan plot from gene-based test by MAGMA using FUMA. There were no genome-wide significant genes (0.05/19369 protein coding genes=2.581×10^{-6}) but the top three genes were: *SH3GL2, PDXDC1* and *RRN3. n*=5018

Supplemental Figure S4: Meta-Analysis of OCD Samples

a) Manhattan plot and b) QQ Plot from the meta-analysis of three OCD case/control cohorts: IOCDF/OCGAS sample, CHOP and Toronto/Michigan Imaging Imaging Genomics Study (Total cases: 3369; total controls: 8611)

Supplemental Figure S5: Meta-Analysis of OC Traits and OCD Samples

Meta-analysis of TOCS total score from the Spit for Science sample (n=5018) with the three OCD/case control cohorts IOCDF/OCGAS sample, CHOP and Toronto/Michigan Imaging Genomics Study (Total cases: 3369; total controls: 8611) using a modified sample-size based weighting meta-analysis that adjusts for SNP heritability (a – Manhattan plot, b – QQ plot) or standard weighting of sample sizes (c – Manhattan plot, d – QQ plot).

Table S1: LD-Independent Top Loci in GWAS of OC Traits in Spit for Science

SNP	A1/A2	A1 Freq	AR2	BETA	SE	P	Gene	Closest Gene(s)
rs7856850	A/C	0.217	1	0.140	0.025	2.48E-08	*PTPRD*	
rs2182089	A/T	0.450	0.94	-0.110	0.021	1.88E-07	*SH3GL2*	
rs691956	A/G	0.406	0.93	0.111	0.022	3.26E-07		*TMEM45B*
rs3754673	C/A	0.121	0.82	-0.170	0.034	7.79E-07	*NPAS2*	
rs9491565	G/T	0.328	0.96	0.109	0.022	1.24E-06	*LOC101928096*	
rs5860287	A/AT	0.547	0.84	0.108	0.023	1.62E-06	*GRID2*	
rs9586307	G/A	0.020	0.72	-0.400	0.084	1.85E-06		*AL136524.1*
rs16997926	T/C	0.030	0.6	0.372	0.078	2.14E-06	*CLDN14*	
rs200674936	A/G	0.711	0.77	-0.122	0.026	2.88E-06		*AC126763.1*
rs2663961	A/G	0.081	0.86	-0.187	0.040	2.96E-06	*PPM1H*	
rs115453328	A/T	0.022	0.8	0.352	0.076	3.74E-06	*HHAT*	
rs59647576	T/C	0.166	0.93	-0.133	0.029	3.76E-06	*NEK11*	
rs8058777	G/A	0.765	0.75	-0.124	0.027	5.37E-06		*RP11-467J12.2/RP11-467J12.3*
rs62399429	A/G	0.152	1	0.131	0.029	5.49E-06	*MUC22*	
rs151001187	T/C	0.017	0.79	-0.389	0.087	8.96E-06	*SGMS2*	

Table S2: Replication of den Braber et al. 2016 in Spit for Science TOCS GWAS

				Den Braber 2016 Results		Spit for Science Results				
**snp**	**chr**	**bp**	**Ref Allele**	**Beta**	**p-value**	**beta**	**se**	**p**	**af**	**Result**
rs60588302	9	7900777	C	1.1278	6.44E-06	0.1118	0.0500	0.0255	0.0542	Replicated
rs11671119	19	19286077	C	1.2036	4.11E-07	0.0206	0.0589	0.7259	0.0401	Not replicated, same direction
rs11658311	17	17470526	C	0.7719	6.50E-06	0.0232	0.0454	0.6089	0.0558	Not replicated, same direction
rs17024030	4	96399606	G	0.7155	6.07E-06	0.0470	0.0468	0.3148	0.0644	Not replicated, same direction
rs17384439	4	96424680	T	0.8323	1.59E-06	0.0438	0.0499	0.3803	0.0507	Not replicated, same direction
rs2837096	21	40978013	G	0.747	2.18E-06	0.0281	0.0435	0.5186	0.0741	Not replicated, same direction
rs4818048	21	40908952	C	0.8956	4.24E-07	0.0204	0.0432	0.6358	0.0674	Not replicated, same direction
rs4818049	21	40910464	G	0.8557	1.03E-06	0.0235	0.0430	0.5846	0.0687	Not replicated, same direction
rs4818050	21	40910600	C	0.8725	6.37E-07	0.0211	0.0434	0.6259	0.0663	Not replicated, same direction
rs4818052	21	40912745	A	0.8475	2.64E-06	0.0228	0.0435	0.6006	0.0667	Not replicated, same direction
rs74276709	21	40913995	A	0.8226	5.85E-06	0.0119	0.0436	0.7857	0.0638	Not replicated, same direction
rs77460585	5	1.01E+08	G	0.8668	4.45E-06	0.0130	0.0560	0.8162	0.0403	Not replicated, same direction
rs77615161	21	40911050	G	0.8541	1.30E-06	0.0245	0.0432	0.5706	0.0677	Not replicated, same direction
rs77959192	21	40911027	C	0.8707	7.36E-07	0.0207	0.0435	0.6344	0.0660	Not replicated, same direction
rs79219884	21	40899981	A	0.8467	6.40E-06	0.0388	0.0473	0.4119	0.0575	Not replicated, same direction
rs8100480	19	19299079	C	1.4095	2.56E-08	0.0135	0.0525	0.7976	0.0512	Not replicated, same direction
rs9520326	13	1.08E+08	T	-0.4326	6.37E-06	-0.0364	0.0217	0.0932	0.4149	Not replicated, same direction
rs999719	22	34264838	T	0.4442	5.33E-06	0.0240	0.0226	0.2870	0.3084	Not replicated, same direction
rs581043	3	62830115	C	0.4358	5.26E-06	-0.0371	0.0236	0.1164	0.6983	Not replicated; opposite direction

Note: reference allele was the same in both samples

These SNPS were the only ones reported in den Braber et al., 2016

Figure S1

Spit for Science Genotyped Samples: 5,837
5,645 HCE (538,448 SNPs)
192 OMNI (1,140,419 SNPs)

Quality Control Step 1: Samples
Sex Mismatch [71]
Call Rate < 97% [17]
Heterozygosity [23]
Removed: n = 93

Quality Control Step 1: SNPs (HCE; OMNI)
Call Rate < 97% [10,636; 138,353]
Hardy-Weinberg [3,403; 1,342]
Duplicated SNPs [7,269; 87,221]
Removed: n = 19,182; 214,978

Quality Control Step 2: Samples
Reported Non-Caucasian [187]
Known Sex Aneuploidy [6]
No standardized TOCS score [129]
Reported diagnosis of ASD [112]
OMNI1 genotyped on HumanCoreExome [9]
Removed: n = 430

Imputation (32,069,643 SNPs)

Quality Control Step 3: Samples
Relatedness (264)
Non-Caucasian/PCA (32)
Removed: n = 296

Quality Control Step 3: SNPS2
MAF ≤ 1% (22,248,184)
AR2 ≤ 0.6 & MAF > 1% (1,951,125)
Removed: n = 24,199,309

Final Sample: 5,018
4,867 HumanCoreExome
151 OMNI1
7,870,334 imputed SNPs

Figure S2

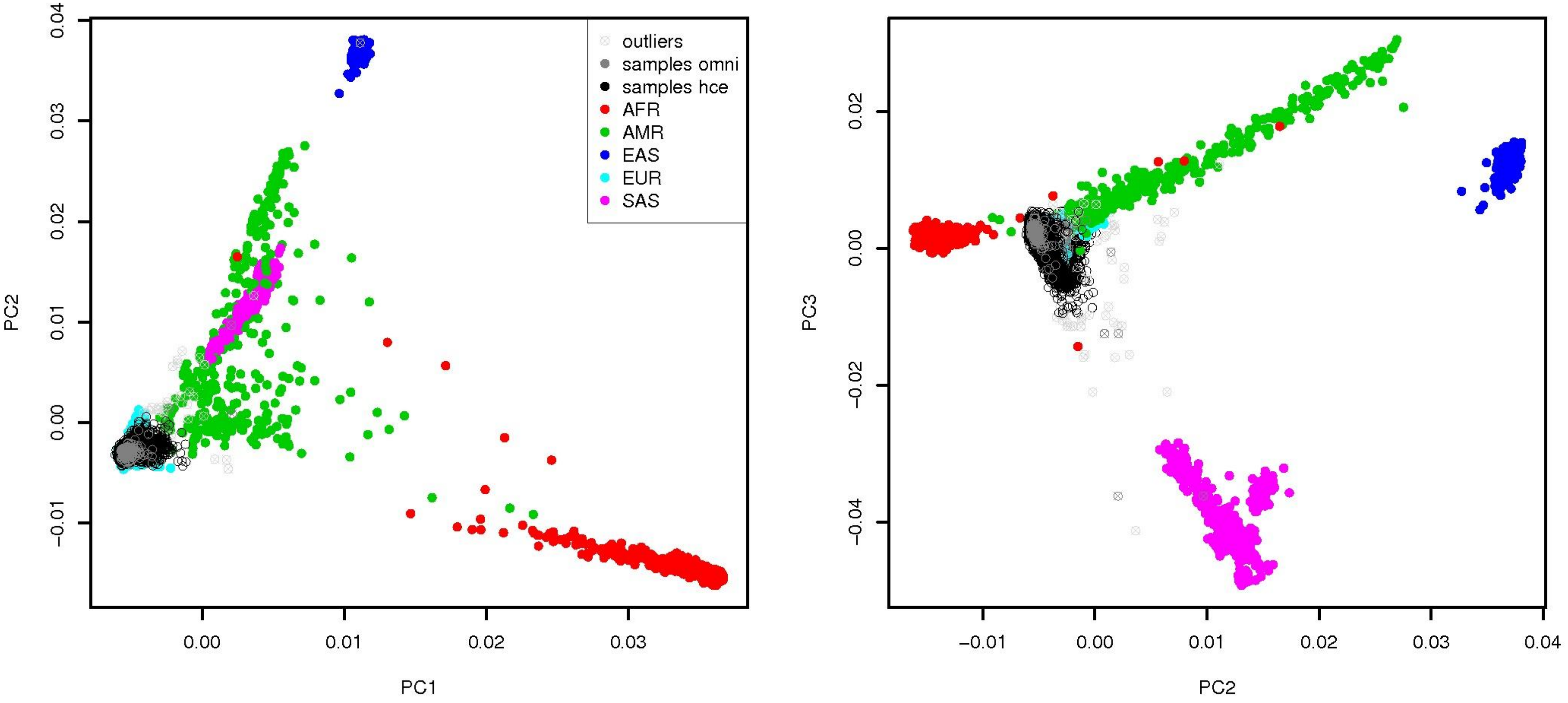
outliers
samples omni
samples hce
AFR
AMR
EAS
EUR
SAS
PC1
PC2
PC3
0.00
0.01
0.02
0.03
0.04
−0.01
−0.02
−0.04

Figure S3

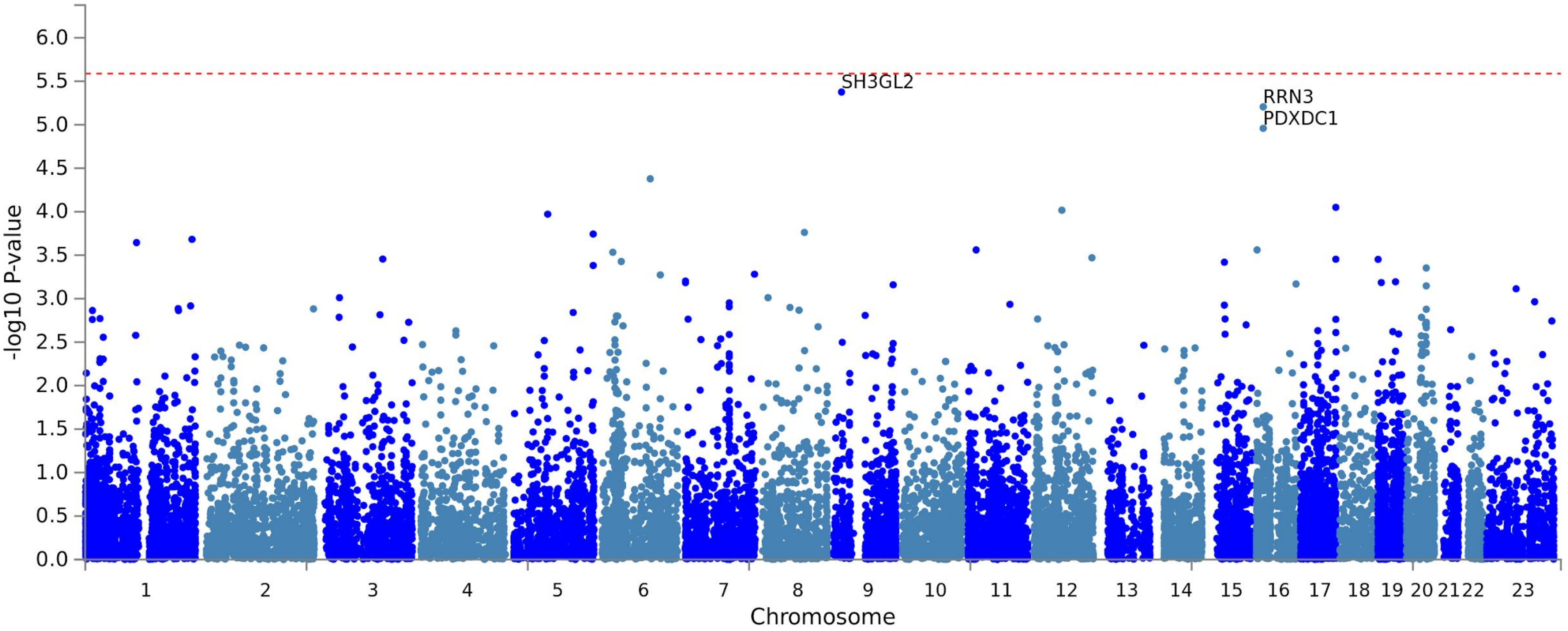

SH3GL2
RRN3
PDXDC1
-log10 P-value
0.0
0.5
1.0
1.5
2.0
2.5
3.0
3.5
4.0
4.5
5.0
5.5
6.0
1
2
3
4
5
6
7
8
9
10
11
12
13
14
15
16
17
18
19
20
21
22
23
Chromosome

Figure S4

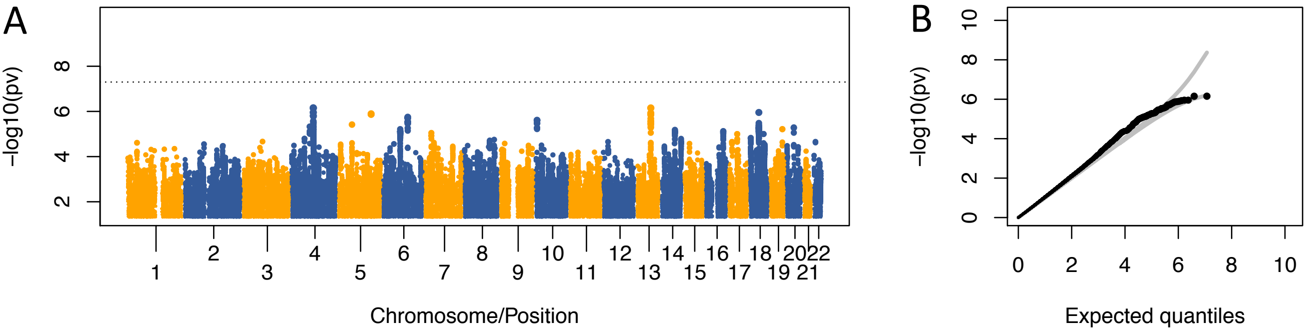
A
B
-log10(pv)
8
6
4
2
1
2
3
4
5
6
7
8
9
10
11
12
13
14
15
16
17
18
19
20
21
22
Chromosome/Position
10
0
Expected quantiles

Figure S5

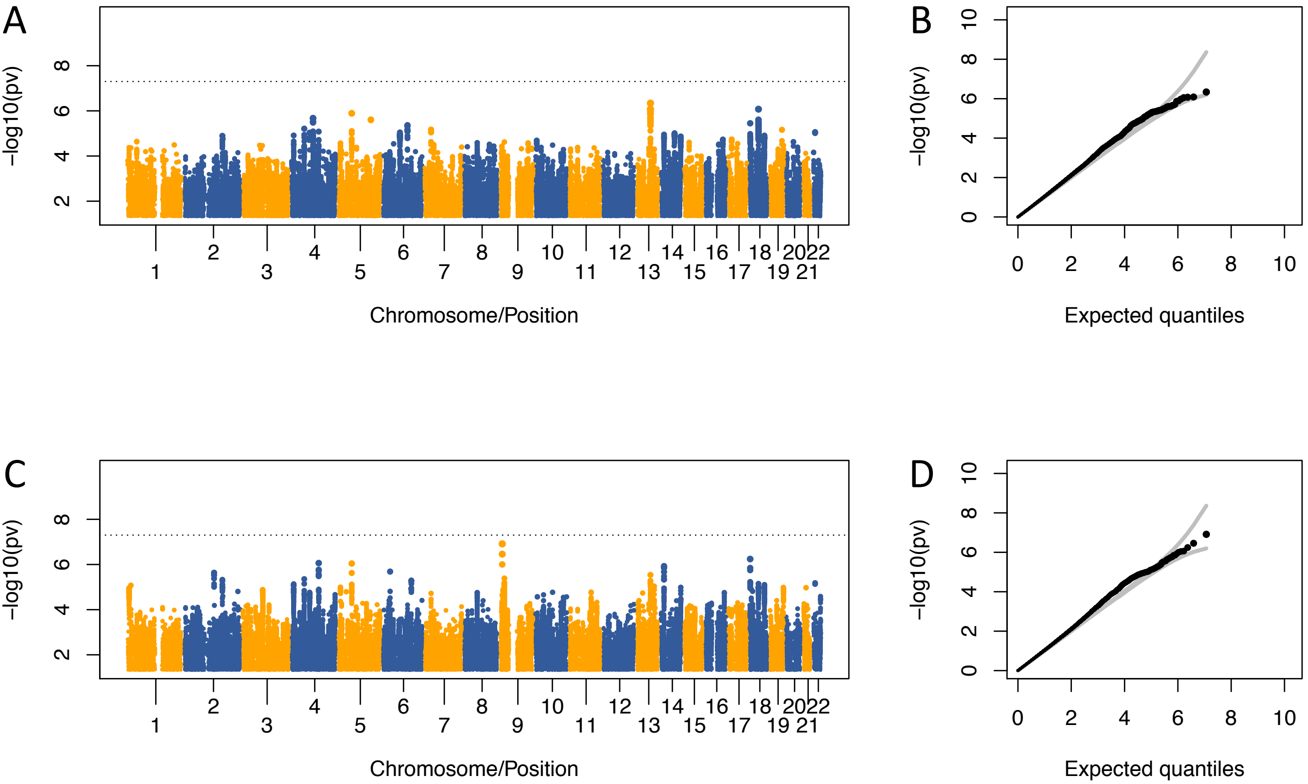
A
B
C
D
-log10(pv)
Chromosome/Position
Expected quantiles
1 2 3 4 5 6 7 8 9 10 11 12 13 14 15 16 17 18 19 20 21 22
